# HEARTLAND Protocol: A Tiered Clinical Implementation Toolkit for Primary Care-Led Heart Failure Management in Rural and Resource-Limited Settings

**DOI:** 10.7759/cureus.104817

**Published:** 2026-03-07

**Authors:** Vicky Muller Ferreira

**Affiliations:** 1 Cardiology, Universidade do Estado do Rio de Janeiro, Rio de Janeiro, BRA

**Keywords:** guideline-directed medical therapy, health disparities, heart failure, hfpef, implementation science, remote patient monitoring, rural health, sglt2 inhibitors, task shifting, telemedicine

## Abstract

Despite decades of evidence-based heart failure (HF) guidelines, no published implementation protocol provides a comprehensive, operational framework for primary care-led HF management specifically designed for rural and resource-limited settings in the United States. Existing quality improvement programs, such as Get With The Guidelines-Heart Failure, identify performance targets but do not provide step-by-step clinical protocols for achieving them in settings with minimal staffing, no specialist access, and limited digital infrastructure. Established HF risk scores similarly omit rural-specific determinants of outcome, including distance to cardiology care and social support, despite robust evidence of their prognostic relevance. The HEARTLAND Protocol (Heart failure Evidence-based Access in Rural Treatment, Linking Advanced Network Delivery) addresses these gaps through a clinical implementation toolkit comprising eight integrated modules that cover the care continuum from hospital admission through long-term management. Developed through a targeted narrative review of clinical evidence published between 2018 and 2025 and informed by the Consolidated Framework for Implementation Research, the protocol synthesizes validated interventions into a tiered structure designed for deployment across varying resource levels. Key features include a three-tier implementation framework accommodating settings from severely constrained critical access hospitals to well-resourced regional centers, a dual-track system supporting both digital and analog monitoring pathways validated by the Hozhó Trial, a pharmacoeconomic generic bridge pathway ensuring that no patient remains untreated due to cost barriers, and a supplementary risk score incorporating rural-specific variables not captured by existing prognostic instruments. The protocol distinguishes between established guideline-supported evidence, emerging trial findings, and pragmatic clinical heuristics, maintaining transparency about evidence strength and methodological limitations throughout. HEARTLAND provides a practical, adaptable framework to extend evidence-based HF care to underserved communities while preserving scientific rigor and positioning facilities for emerging value-based payment models.

## Introduction

Heart failure (HF) represents one of the most significant public health challenges in the United States, imposing an enormous and growing burden on patients, healthcare systems, and the economy. According to the American Heart Association's 2025 Heart Disease and Stroke Statistics Update, approximately 6.7 million Americans currently live with HF, with projections indicating that the prevalence will nearly double by 2050 [1]. The condition accounts for over one million hospitalizations annually and represents the leading cause of hospitalization among Medicare beneficiaries, with current annual costs reaching hundreds of billions of dollars and projections suggesting substantial continued growth over the coming decades. The 2022 American Heart Association/American College of Cardiology/Heart Failure Society of America (AHA/ACC/HFSA) Guideline for Management of Heart Failure established comprehensive, evidence-based recommendations for pharmacological and nonpharmacological treatment across the full spectrum of left ventricular ejection fraction (LVEF) [2]. However, the gap between guideline publication and real-world implementation remains vast.

Rural and underserved populations bear a disproportionate share of this burden, and the disparities are widening rather than narrowing. A population-based study demonstrated that rurality is independently associated with significantly higher risk of incident HF even after adjusting for traditional cardiovascular risk factors, with rural residents experiencing substantially higher cardiovascular death rates compared to urban counterparts [3]. Access to specialist care compounds these geographic disparities: the vast majority of rural counties in the United States lack any practicing cardiologist, with a mean distance to cardiology care reaching 87 miles in counties without a cardiologist compared to 16 miles in counties with one [4]. These findings collectively demonstrate that the HF epidemic is not uniformly distributed and that rural populations face a convergence of higher disease burden, accelerating mortality trends, and severely constrained access to expert management.

The evidence base for effective HF management continues to strengthen. For HF with reduced ejection fraction (HFrEF), guideline-directed medical therapy (GDMT) with quadruple therapy - comprising angiotensin receptor-neprilysin inhibitors (ARNI), beta-blockers, mineralocorticoid receptor antagonists (MRA), and sodium-glucose cotransporter-2 inhibitors (SGLT2i) - is associated with substantial mortality reduction [5]. For HF with preserved ejection fraction (HFpEF) - historically an area with limited disease-modifying therapies - recent trials have transformed the treatment landscape. The EMPagliflozin outcomE tRial in Patients With chrOnic heaRt Failure With Preserved Ejection Fraction (EMPEROR-Preserved) trial demonstrated that empagliflozin reduces the combined risk of cardiovascular death or hospitalization for HF in patients with LVEF >40% [6], and the Dapagliflozin Evaluation to Improve the Lives of Patients with Preserved Ejection Fraction Heart Failure (DELIVER) trial confirmed these benefits with dapagliflozin across a broad HFpEF population [7], supporting a Class IIa recommendation for SGLT2i in HFpEF per the 2022 AHA/ACC/HFSA guideline [2]. Beyond SGLT2i, the Finerenone Trial to Investigate Efficacy and Safety Superior to Placebo in Patients With Heart Failure (FINEARTS-HF) trial demonstrated that finerenone reduces cardiovascular death and HF events in patients with LVEF >40% [8]. The Semaglutide Treatment Effect in People with obesity and HFpEF (STEP-HFpEF) trial showed that semaglutide improves symptoms and functional capacity in patients with the obesity phenotype of HFpEF [9]. While these findings represent meaningful advances, guidelines are still being updated to incorporate this evidence, and clinicians should be aware of the evolving nature of recommendations for HFpEF.

Despite this robust evidence base, implementation remains deficient. Registry data indicate that, among eligible outpatients with HFrEF, fewer than one in four receive all recommended medication classes and that fewer than 1% simultaneously achieve target doses [5]. This implementation gap is particularly pronounced in resource-limited settings. The AHA's Get With The Guidelines-Heart Failure (GWTG-HF) program, now in its 20th year, has enrolled over 600 hospitals and demonstrated measurable quality improvement [10]. Following the AHA's Presidential Advisory on Rural Health [11], the AHA launched the Rural Healthcare Outcomes Accelerator in 2022, providing up to 700 rural hospitals with no-cost GWTG-HF access. Even international registries such as the European Society of Cardiology Heart Failure Long-Term Registry (ESC-HF-LT), which tracks outcomes across multiple countries, include no rural-specific adaptation or operational guidance for resource-limited settings [12]. However, these programs function as quality benchmarking and performance improvement platforms rather than operational clinical protocols - they identify what should be done but do not provide a step-by-step implementation framework for how to do it in settings with one to two nurses, no pharmacist, no specialist, and limited broadband. To our knowledge, no published implementation protocol provides a comprehensive, operational framework for primary care-led HF management specifically designed for rural and resource-limited settings in the United States. This gap is the problem Heart failure Evidence-based Access in Rural Treatment, Linking Advanced Network Delivery (HEARTLAND) addresses.

Recent evidence has demonstrated the feasibility of delivering quality HF care in underserved settings through innovative approaches. The Hozhó Trial, conducted among American Indians in the rural Navajo Nation, achieved substantial absolute increases in GDMT class addition using telephone-based optimization [13]. Critically, this intervention used voice telephone calls rather than digital applications, demonstrating that low-technology approaches can be effective in populations with limited broadband access. The Telemedical Interventional Management in Heart Failure II (TIM-HF2) trial demonstrated mortality reduction with structured remote monitoring, with benefits most pronounced among patients living farthest from cardiology care [14]. These studies challenge the assumption that high-quality HF management requires resource-intensive infrastructure and suggest that pragmatic, technology-appropriate interventions can meaningfully improve outcomes in underserved communities.

Federal policy developments reinforce the urgency of this work. The Centers for Medicare and Medicaid Services (CMS) has announced the Ambulatory Specialty Model (ASM), a value-based payment program for HF management with implementation anticipated in 2027 [15]. The AHA's Presidential Advisory on Rural Health called for the development of healthcare delivery models adapted to rural constraints [11]. The AHA Scientific Statement on Implementation Science to Achieve Equity in Heart Failure Care specifically identified the need for implementation frameworks that address geographic and socioeconomic barriers [16]. HEARTLAND responds directly to these calls.

This paper presents the HEARTLAND Protocol, a clinical implementation toolkit synthesizing validated interventions into a tiered protocol designed for deployment across varying resource levels. The protocol distinguishes between established and emerging evidence, incorporates honest acknowledgment of methodological limitations, and provides operational flexibility for settings from well-resourced regional centers to severely constrained rural clinics.

## Technical report

Methods

Evidence Synthesis Approach

We conducted a targeted narrative review of clinical evidence for HF management interventions. This approach was selected because HEARTLAND is an implementation framework, not a systematic review. We searched PubMed and Cochrane Library for publications from January 2018 through December 2025, identifying over 60 relevant publications, including randomized controlled trials demonstrating mortality or hospitalization benefit, interventions validated in underserved or rural populations, protocols demonstrating feasibility for non-specialist delivery, and recent evidence for HFpEF therapeutics. We additionally reviewed authoritative guidelines from the AHA, HFSA, and ACC, including the foundational 2022 AHA/ACC/HFSA HF management guideline [2]. Protocol development was further informed by publicly reported quality metrics from the AHA Rural Healthcare Outcomes Accelerator program, Hospital Readmissions Reduction Program (HRRP) data for critical access hospitals, and operational workflows described in rural health literature from Federally Qualified Health Centers (FQHCs) and Rural Health Clinics.

Conceptual Framework

The protocol structure was informed by the Consolidated Framework for Implementation Research (CFIR) [17], recognizing that successful intervention translation requires attention to intervention characteristics, setting factors, and the implementation process. Risk stratification was structured around the AHA's 2023 Presidential Advisory on cardiovascular-kidney-metabolic (CKM) syndrome [18].

Tiered Implementation Design

Acknowledging that resource availability varies dramatically across settings, we developed three implementation tiers. Tier 1 (Minimal) is designed for severely constrained settings, such as a small critical access hospital with one to two nurses. Tier 2 (Standard) targets moderate-resource settings, such as an FQHC or community hospital. Tier 3 (Advanced) is intended for well-resourced settings, such as a regional referral center. Each tier specifies realistic expectations, recognizing that incremental improvement from baseline - not achievement of all targets - constitutes success in resource-limited settings.

Pharmacoeconomic Integration

We developed a "generic bridge" pathway, ensuring that no patient remains untreated while optimal agents are pursued. This pathway explicitly authorizes generic alternatives as appropriate therapy, not merely a compromise. The rationale is rooted in the principle that any GDMT is superior to no GDMT, and that cost should never be a reason for therapeutic inaction.

Risk Score Development Rationale

Established HF risk scores were evaluated for applicability to rural primary care settings. The Meta-Analysis Global Group in Chronic Heart Failure (MAGGIC) score uses 13 clinical variables to predict one- to three-year mortality but does not incorporate distance to care, social support, or rurality [19]. The Seattle Heart Failure Model (SHFM) uses over 24 variables for survival prediction, but its complexity limits primary care feasibility, and it similarly omits geographic and social determinants [20]. Importantly, perceived social isolation has been independently associated with a 3.74-fold increase in mortality among HF patients [21], and social deprivation indices are significantly associated with HF readmission [22], yet no established risk score incorporates these variables. We therefore developed a pragmatic supplementary heuristic - the HEARTLAND Risk Score - that layers rural-specific variables (distance to cardiology, social support) onto established clinical risk factors. Weights were assigned based on relative effect sizes reported in the cited observational literature and represent a pragmatic consensus pending formal derivation [3,4,21,22]. This score is explicitly positioned as a clinical decision-support tool to guide monitoring intensity, not as a validated prognostic instrument competing with MAGGIC or SHFM. A comparison of risk score characteristics is provided (Table 1).

**Table 1:** Comparison of the Established Heart Failure Risk Scores With the HEARTLAND Risk Score HEARTLAND: Heart failure Evidence-based Access in Rural Treatment, Linking Advanced Network Delivery; GWTG-HF: Get With The Guidelines-Heart Failure; MAGGIC: Meta-Analysis Global Group in Chronic Heart Failure; SHFM: Seattle Heart Failure Model

Characteristic	MAGGIC	GWTG-HF	SHFM	HEARTLAND
Number of Variables	13	7	24+	10
Prediction Target	1-3 year mortality	In-hospital mortality	1-5 year survival	Readmission risk and monitoring intensity
Intended Setting	All settings	Hospital	All settings	Rural and resource-limited
Includes Distance to Care	No	No	No	Yes (+1 point if >50 miles)
Includes Social Support	No	No	No	Yes (+1 point if limited)
Includes Rurality Markers	No	No	No	Yes (distance + social support)
Validated	Yes (derivation: 39,372 patients, 30 studies)	Yes (derivation: 39,783 patients)	Yes (derivation: 1,125 patients)	No (pragmatic heuristic; validation planned)
Feasibility in Primary Care	Moderate (requires 13 clinical variables)	Low (designed for hospital use)	Low (requires 24+ variables, complex)	High (10 variables, bedside calculation)

Transparency Regarding Evidence Strength

Throughout the protocol, we distinguish between three categories of evidence. Established evidence refers to interventions with strong guideline support, such as SGLT2i for HFrEF as endorsed with a Class I recommendation by the 2022 AHA/ACC/HFSA guideline [2], and SGLT2i for HFpEF with a Class IIa recommendation supported by the EMPEROR-Preserved [6] and DELIVER [7] trials. Emerging evidence refers to recent trial findings not yet fully incorporated into guidelines, such as finerenone for HFpEF based on the FINEARTS-HF trial [8]. Pragmatic heuristics refer to tools developed for clinical utility without formal statistical validation, such as the HEARTLAND Risk Score. This three-tiered labeling system helps clinicians understand the confidence level behind each recommendation and make appropriately calibrated clinical decisions.

Results

The resulting HEARTLAND Protocol comprises eight integrated modules addressing the care continuum from hospital admission through long-term management. Each module targets a specific aspect of HF care delivery, and all are designed with tiered implementation options to accommodate varying resource levels.

Module 1: Risk Stratification

Risk stratification integrates CKM syndrome staging with a supplementary HEARTLAND Risk Score. The CKM framework stages patients from Stage 0 (no risk factors) through Stage 4 (clinical cardiovascular disease), with Stage 4 associated with a substantial reduction in life expectancy [18]. The HEARTLAND Risk Score (0-18 points) incorporates 10 factors associated with readmission and amenable to intervention: age, prior hospitalization, renal function, natriuretic peptides, hemodynamics, diabetes, ejection fraction, CKM stage, distance to cardiology, and social support. Critically, the final two variables - distance to cardiology care (>50 miles) and limited social support - address the rural-specific determinants that established scores omit. The complete scoring reference is provided for clinical use (Table 2).

**Table 2:** HEARTLAND Risk Score Reference HEARTLAND: Heart failure Evidence-based Access in Rural Treatment, Linking Advanced Network Delivery Maximum Score: 18 points. LOW (0-4): Standard; MODERATE (5-8): Enhanced Bundle; HIGH (≥9): Intensive Bundle

Risk Factor	Points
Age ≥75 years	+2
Prior heart failure (HF) hospitalization within 6 months	+3
Estimated glomerular filtration rate (eGFR) <45 mL/min/1.73 m²	+2
B-type natriuretic peptide (BNP) ≥500 pg/mL or N-terminal pro-B-type natriuretic peptide (NT-proBNP) ≥1500 pg/mL	+2
Systolic blood pressure (BP) <100 mmHg at admission	+2
Diabetes mellitus	+1
Left ventricular ejection fraction (LVEF) <30%	+2
Cardiovascular-kidney-metabolic (CKM) Stage 3 or 4	+2
Distance to cardiology care >50 miles	+1
Lives alone or limited social support	+1

It is important to note that the HEARTLAND Risk Score is a pragmatic heuristic designed to identify patients who may benefit from intensified monitoring and support. Weights were assigned based on relative effect sizes reported in the cited observational literature and represent a pragmatic consensus pending formal derivation [3,4,21,22]; the score has not been statistically validated through derivation and validation cohorts and should be used as a clinical decision-support tool that supplements - rather than replaces - validated prognostic instruments, such as the MAGGIC score [19]. Formal derivation and validation using registry data represent a planned next step in this research program.

Module 2: GDMT Optimization with 2025 Pharmacology

**Figure 1:**
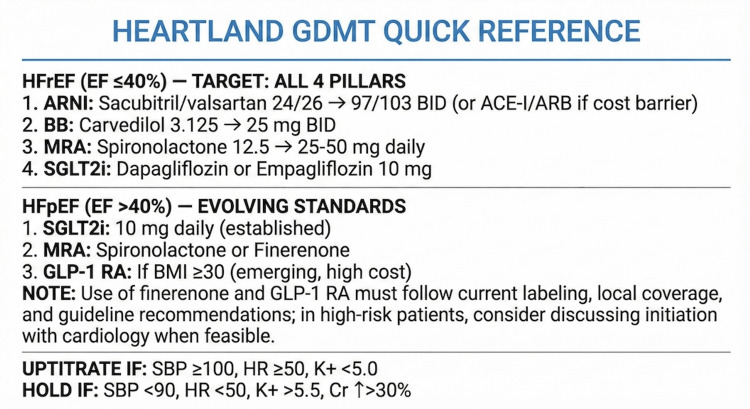
Guideline-Directed Medical Therapy (GDMT) Quick Reference Card ARNI: angiotensin receptor-neprilysin inhibitors; HFpEF: HF with preserved ejection fraction; SGLT2i: sodium-glucose cotransporter-2 inhibitors; MRA: mineralocorticoid receptor antagonists; GLP-1 RAs: glucagon-like peptide-1 receptor agonists; HFrEF: HF with reduced ejection fraction

The protocol embraces the philosophy that "some therapy is better than no therapy," balancing evidence-based medicine with practical constraints. For HFrEF (LVEF ≤40%), the protocol targets initiation of all four GDMT pillars: angiotensin receptor-neprilysin inhibitors (ARNI) (or angiotensin-converting enzyme inhibitor/angiotensin receptor blocker (ACE-I/ARB)), beta-blocker, MRA, and SGLT2i. For Tier 1 facilities, the minimum standard is at least two classes initiated before discharge. A visual GDMT quick reference card is provided to support bedside decision-making (Figure 1).

For HFpEF (LVEF >40%), the protocol incorporates evolving evidence with appropriate context. SGLT2 inhibitors carry a Class IIa recommendation per the 2022 AHA/ACC/HFSA guideline [2], supported by the EMPEROR-Preserved [6] and DELIVER [7] trials, and represent an evolving standard of care. MRA remain an established option, with spironolactone as the traditional agent; finerenone, based on the FINEARTS-HF trial [8], represents emerging evidence that may become preferred in appropriate patients, particularly those with concurrent chronic kidney disease (CKD), with appropriate laboratory monitoring (baseline potassium <5.0 mEq/L and eGFR ≥25 mL/min/1.73 m² per FINEARTS-HF inclusion criteria). Clinicians should be aware that guideline updates incorporating FINEARTS-HF data are ongoing. Glucagon-like peptide-1 receptor agonists (GLP-1 RAs), supported by the STEP-HFpEF trial [9], may benefit patients with the obesity phenotype (BMI ≥30), though this is best conceptualized as obesity therapy with cardiovascular benefits rather than primary HF therapy, and cost constraints (approximately $1,000 per month) and supply issues remain significant barriers.

Non-pharmacological management is integrated throughout the protocol, including structured guidance for sodium restriction (<2,000 mg/day), physical activity with cardiac rehabilitation referral as a Class I recommendation, alcohol limitation, tobacco cessation, and weight management. Pharmacoeconomic navigation is addressed through the "generic bridge" pathway (estimated at approximately $15 per month based on $4 generic programs at major retail pharmacies), which ensures that patients receive foundational therapy - including generic ACE-I/ARB, beta-blocker, and spironolactone - while optimal agents are pursued through patient assistance programs (PAP). The protocol's financial navigation tracker provides a structured tool for monitoring insurance status, therapy optimization, and PAP applications (Table 3). The protocol explicitly states: "generic therapy is superior to no therapy. Never delay treatment while waiting for paperwork."

**Table 3:** HEARTLAND Financial Navigation Tracker HEARTLAND: Heart failure Evidence-based Access in Rural Treatment, Linking Advanced Network Delivery

Field	Entry
Patient	(Text field)
Date	(Text field)
Insurance Type	Commercial/Medicare/Medicaid/Uninsured/340B Eligible
Current Therapy	Generic Bridge/Partial Optimal/Full Optimal

Medication	Date Applied	Status (P/A/D)	Approval
-	-	-	-

Module 3: Telephone-Based GDMT Titration

This module adapts methodology validated in the Hozhó Trial [13], which demonstrated that telephone-based interventions can achieve substantial improvements in GDMT prescribing among underserved populations. The protocol specifies two parallel pathways operating as a dual-track execution system. Track A (Digital) employs app-based symptom tracking, Bluetooth-enabled devices, and automated data transmission. Track B (Analog) relies on voice telephone calls, paper symptom diaries, and manual data entry. Both tracks follow identical clinical algorithms, differing only in data collection method. The Hozhó Trial's success with voice telephone validates that analog pathways are not merely fallback options but effective primary interventions. The track assignment form guides clinicians through a structured assessment of patient digital literacy, connectivity, and resource availability to determine the most appropriate monitoring pathway (Table 4).

**Table 4:** HEARTLAND Track Assignment Form HEARTLAND: Heart failure Evidence-based Access in Rural Treatment, Linking Advanced Network Delivery

Assessment Item	Response
Patient Name	(Text field)
Medical Record Number (MRN)	(Text field)
Risk Score	(Text field)
Smartphone with reliable connectivity?	Yes/No
Comfortable using apps?	Yes/No
Reliable telephone access?	Yes/No
Track Assignment	Track A (Digital)/Track B (Analog)/Hybrid
Implementation Tier	Tier 1/Tier 2/Tier 3
Equipment Provided	Blood pressure (BP) cuff/Scale/Paper diary
Signature and Date	(Text field)

Module 4: Structured Discharge Transitions

**Figure 2:**
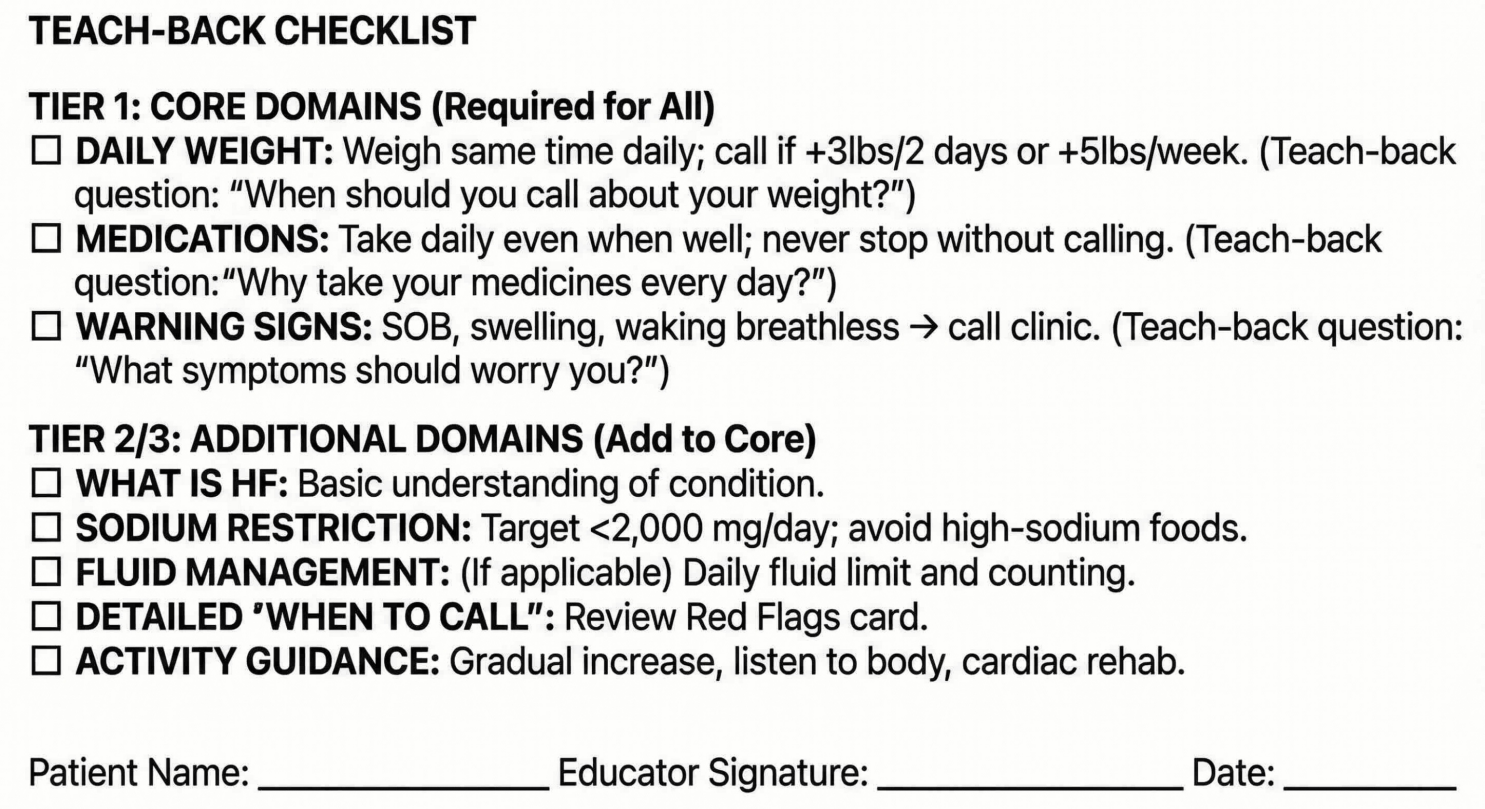
Teach-Back Checklist

The discharge bundle comprises case management assessment, teach-back education, medication reconciliation, and bedside delivery [23] and scheduled follow-up. Transitional care interventions have demonstrated significant reductions in HF readmissions across multiple studies [24]. The teach-back methodology, which requires patients to explain back information in their own words, ensures comprehension rather than mere information delivery. A structured teach-back checklist guides staff through domain-specific education sessions, with Tier 1 facilities focusing on three core domains (daily weight, medications, warning signs) and Tier 2/3 facilities implementing eight comprehensive domains (Figure 2).

Recognizing nursing constraints in rural facilities, the protocol specifies a task-shifting framework with tiered execution. Clinical decisions remain with licensed clinicians, but data collection and education delivery can be distributed across the available workforce. For facilities without community health workers (CHWs), alternatives include registered nurses or medical assistants (RN/MA) using standardized scripts, family caregiver training, and automated interactive voice response (IVR) systems.

Module 5: Remote Patient Monitoring

**Figure 3:**
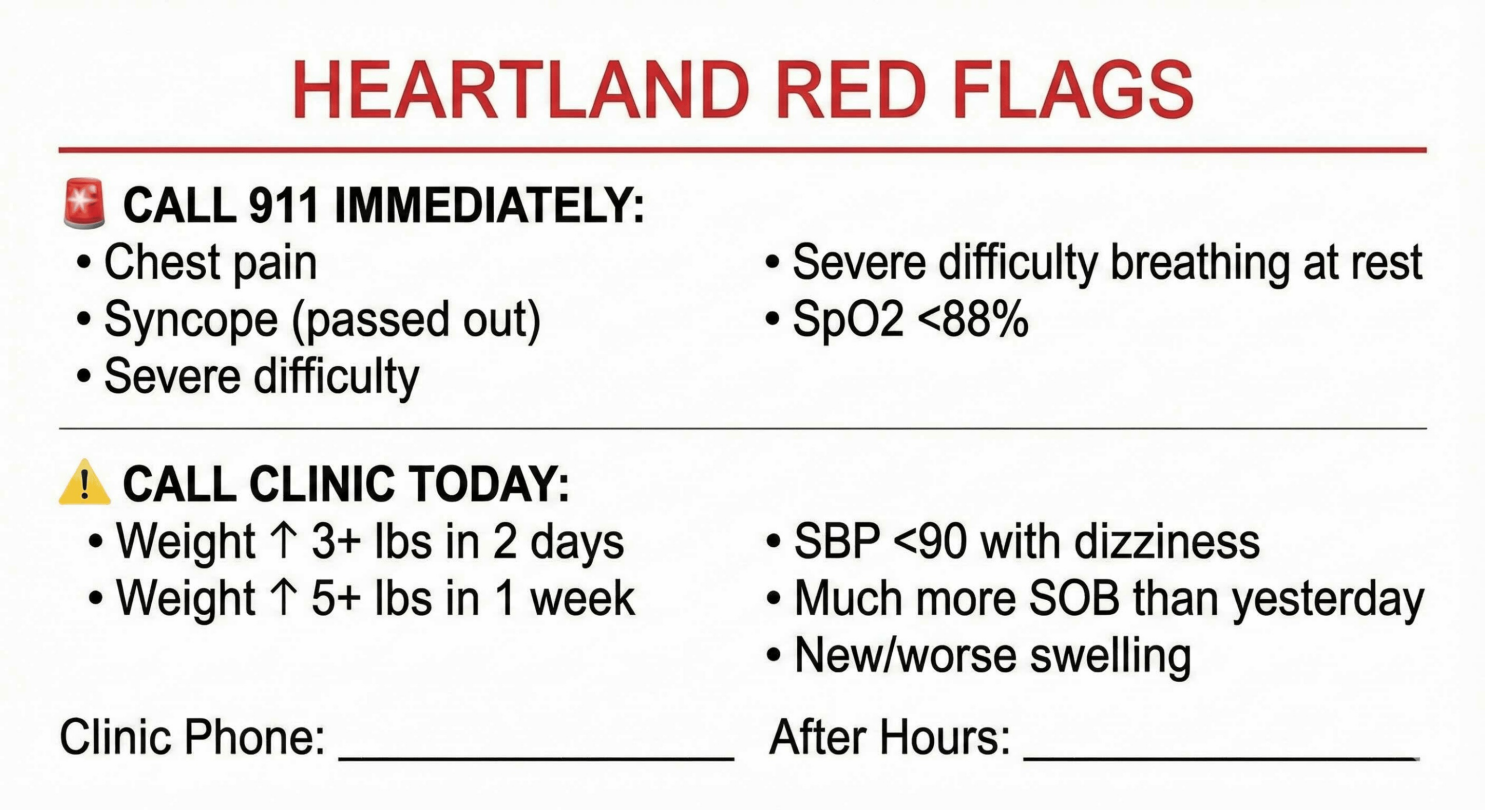
Red Flags Alert Card SBP: systolic blood pressure; SOB: shortness of breath; SpO_2_: oxygen saturation

**Figure 4:**
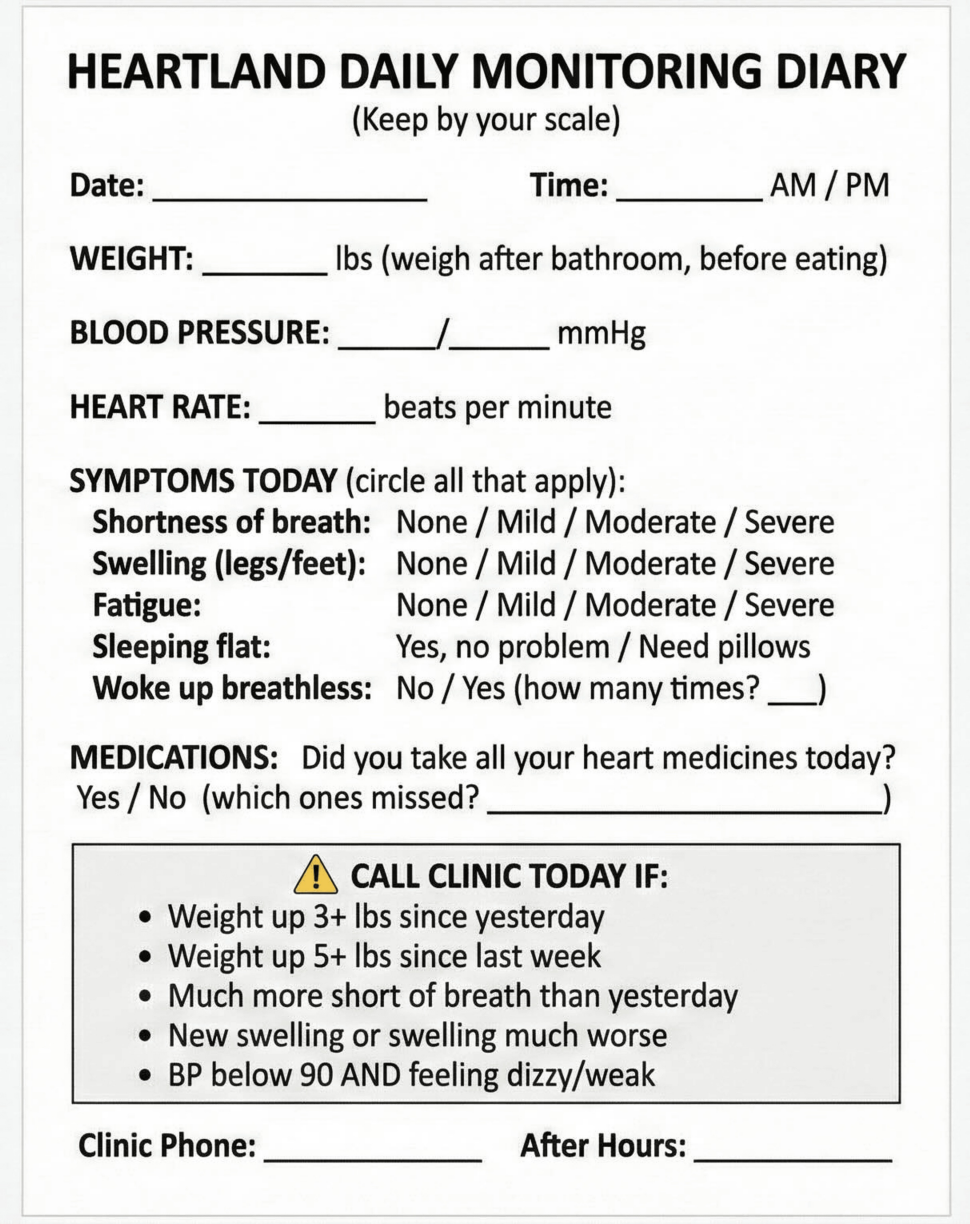
Patient Daily Monitoring Diary

The TIM-HF2 trial demonstrated meaningful mortality reduction with structured remote monitoring, with the greatest benefit among patients living farther from cardiology care [14]. The basic monitoring kit (estimated $50-150) comprises a digital scale, blood pressure (BP) monitor, and pulse oximeter (if indicated). The "human filter" principle requires that all non-emergency alerts pass through a licensed clinician's telephone assessment before emergency department (ED) referral, preventing alarm fatigue and inappropriate utilization. A red flag alert card provides visual guidance for patients and caregivers to identify warning signs requiring immediate clinical contact (Figure 3). Additionally, a patient's daily monitoring diary supports structured self-tracking for patients using the analog pathway (Figure 4).

Financial sustainability is addressed through remote patient monitoring/remote therapeutic monitoring (RPM/RTM) billing codes (99453-99458, 98975-98981), with potential revenue of $150-200 per high-risk patient monthly. These reimbursement mechanisms make remote monitoring economically viable even for resource-constrained practices, offsetting equipment and staffing costs while generating net positive revenue for high-risk patient management.

Module 6: Comorbidity Management

The protocol provides practical guidance for common comorbidities encountered in HF patients, recognizing that multi-morbidity is the rule rather than the exception. Conditions addressed include atrial fibrillation (anticoagulation and rate control), obstructive sleep apnea (screening and continuous PAP (CPAP) referral), iron deficiency (ferritin and transferrin saturation (TSAT) assessment with intravenous iron if indicated), diabetes (leveraging SGLT2i as dual therapy), CKD (dose adjustments and monitoring for hyperkalemia), chronic obstructive pulmonary disease (COPD; noting that beta-blocker concerns are generally overstated and cardioselective agents are usually tolerated), and depression (screening with the Patient Health Questionnaire-9 (PHQ-9) and treatment with selective serotonin reuptake inhibitors (SSRIs), avoiding tricyclic antidepressants (TCAs) due to their negative inotropic effects). A comorbidity quick reference summarizes key considerations, recommended actions, and pitfalls to avoid for each condition (Table 5).

**Table 5:** Comorbidity Quick Reference

Comorbidity	Key Considerations	What to Do	What to Avoid
Atrial Fibrillation	Rate vs rhythm	Rate control, anticoagulation	Stopping anticoagulation
Obstructive Sleep Apnea	Prevalent in heart failure with preserved ejection fraction (HFpEF)	Screen (STOP-BANG), continuous positive airway pressure (CPAP)	Assuming "just heart failure (HF)"
Iron Deficiency	Worsens symptoms	Check ferritin/transferrin saturation (TSAT), intravenous (IV) iron	Oral iron
Diabetes	Sodium-glucose cotransporter-2 inhibitor (SGLT2i) is dual therapy	SGLT2i, metformin, glucagon-like peptide-1 receptor agonist (GLP-1 RA)	Thiazolidinediones
Chronic kidney disease (CKD)	Limits dosing, hyperkalemia	Adjust doses, monitor potassium, finerenone	Avoiding guideline-directed medical therapy (GDMT)
Chronic obstructive pulmonary disease (COPD)	Beta-blocker (BB) concerns overstated	Cardioselective BB (metoprolol)	Withholding BB
Depression	Affects adherence	Screen (Patient Health Questionnaire-9 (PHQ-9)), selective serotonin reuptake inhibitors (SSRIs)	Tricyclic antidepressants (TCAs)
Hypertension	GDMT treats both	GDMT lowers blood pressure (BP), adjust others	Adding medications before GDMT optimization

An advanced HF referral pathway specifies criteria for tertiary referral: LVEF ≤35% despite optimal GDMT, recurrent hospitalizations, need for device evaluation, suspected infiltrative disease, or advanced therapy consideration. Clear referral criteria help primary care teams identify when specialist input is essential while maintaining confidence in managing stable patients independently.

Module 7: Primary Care Coordination

**Figure 5:**
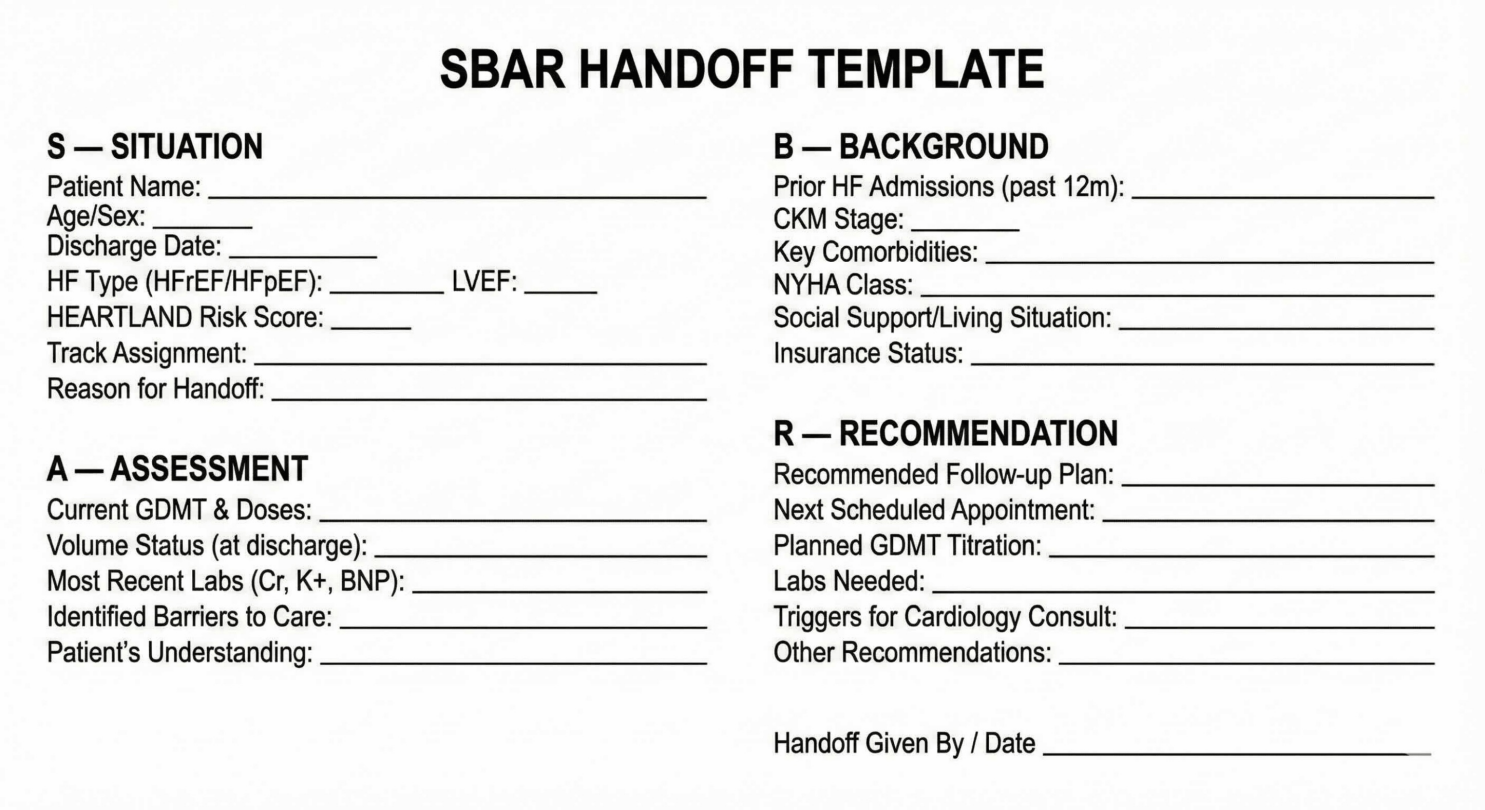
SBAR Handoff Template SBAR: situation-background-assessment-recommendation

Situation-background-assessment-recommendation (SBAR) handoff tools structure communication between hospital and primary care providers (PCPs), ensuring critical clinical information transfers reliably across care transitions. The SBAR handoff template provides a standardized format for documenting patient status, clinical trajectory, and ongoing management needs at the time of care transition (Figure 5). Criteria clarify when cardiology input is needed versus when PCPs can manage independently, reducing unnecessary specialist referrals while ensuring appropriate escalation. Shared medical appointments (SMAs) may substantially increase provider capacity for stable patient management, as a single group session with 8-10 patients effectively replaces an equivalent number of individual encounters of similar duration, enabling group-based education and monitoring that is particularly valuable in settings with limited clinician availability.

Module 8: Implementation Guidance

The three implementation tiers accommodate varying resources across the full spectrum of healthcare settings. Tier 1 (Minimal) focuses on 48-72 hour post-discharge contact, initiation of at least two GDMT classes, analog monitoring, and condensed teach-back, with the target of incremental improvement from baseline. Tier 2 (Standard) encompasses full protocol implementation over 12 months with phased rollout, targeting protocol-specified metrics with quarterly review. Tier 3 (Advanced) encompasses complete implementation, including rapid up-titration of GDMT as supported by the Safety, Tolerability and Efficacy of Rapid Optimization, Helped by NT-proBNP Testing, of Heart Failure Therapies (STRONG-HF) trial [25], which demonstrated that intensive, early optimization of HF therapies reduces the composite of HF rehospitalization and all-cause death [25,26], full RPM capture through digital pathways, SMAs, and regional hub function serving surrounding facilities. Quality metrics are tier-specific, with Tier 1 focusing on contact rates and basic GDMT initiation, and Tier 2/3 targeting more comprehensive goals, including dose optimization and readmission reduction. A comprehensive summary of implementation expectations across all three tiers is provided (Table 6).

**Table 6:** Implementation Tier Summary CKM: cardiovascular-kidney-metabolic

Component	Tier 1 (Minimal)	Tier 2 (Standard)	Tier 3 (Advanced)
Risk Stratification	Score at discharge	Full CKM + Score	Full CKM + Score
Guideline-directed medical therapy (GDMT)	≥2 classes, prioritize sodium-glucose cotransporter-2 inhibitor (SGLT2i) + beta-blocker (BB)	Target all classes in 14 days	Rapid-sequence initiation
Monitoring	Track B (Analog)	Dual-track (A/B)	Track A primary + remote patient monitoring (RPM)
Discharge Education	Condensed teach-back (3 domains)	Full teach-back (8 domains)	Full teach-back
Follow-up	48–72 hour call, 14-day visit	48-hour call, 7-day visit, weekly ×4	48-hour call, 7-day visit, frequent
Staffing	Registered nurse/medical assistant (RN/MA) + physician (MD)	RN champion, MA, PharmD	Full team (RN, PharmD, social worker, community health worker (CHW))
Community health worker (CHW)	Alternative/Family	High-risk only	Full integration
Financial	Generic Bridge	Patient assistance program (PAP) pursuit + Generic bridge	PAP pursuit + Generic bridge

## Discussion

The HEARTLAND Protocol addresses a specific, documented gap in HF care delivery: the absence of a comprehensive, operational implementation protocol designed for primary care-led HF management in rural and resource-limited settings. While quality improvement programs such as GWTG-HF have achieved meaningful improvements in hospital-based care [10], and federal initiatives such as the Rural Healthcare Outcomes Accelerator have expanded access to these programs in rural areas, no published framework provides the step-by-step operational guidance needed to translate HF guidelines into practice at a critical access hospital with two nurses and no specialist on-site.

Positioning Against Existing Implementation Frameworks

The landscape of HF implementation programs includes several important initiatives, each with distinct contributions and limitations. The AHA's GWTG-HF program, now enrolling over 600 hospitals, focuses on quality benchmarking, data registry participation, and performance recognition [10]. The Rural Healthcare Outcomes Accelerator, launched in 2022 in response to the Presidential Advisory, extended GWTG-HF access to rural hospitals and provided program consultants. However, these programs identify performance targets without providing operational clinical protocols for achieving them in resource-constrained environments. The ESC-HF-LT Registry, a multi-country European registry, tracks HF outcomes and treatment patterns across diverse healthcare systems [12], but includes no rural-specific modifications, no guidance for settings without pharmacy support, and no analog monitoring pathways. The AHA Scientific Statement on Implementation Science to Achieve Equity in Heart Failure Care [16] articulated the theoretical framework for addressing disparities but did not produce operational toolkits. HEARTLAND complements these programs by providing the operational layer they lack: specific titration protocols, monitoring algorithms, task-shifting workflows, and pharmacoeconomic navigation tools adapted for resource-variable settings.

The Rural Risk Assessment Gap

Established HF risk scores were developed using clinical variables from tertiary care datasets and do not incorporate determinants of outcome that are disproportionately relevant in rural populations. The MAGGIC score, derived from 39,372 patients across 30 studies, uses 13 clinical variables for mortality prediction [19]. The SHFM incorporates over 24 variables for survival prediction [20]. None of these instruments includes distance to cardiology care, transportation access, social support, or rurality - all of which are independently associated with HF outcomes. Rurality itself is associated with a significantly higher risk of incident HF independent of traditional risk factors [3]. The mean distance to cardiology care reaches 87 miles in counties without a cardiologist [4]. Perceived social isolation is associated with a 3.74-fold increase in HF mortality [21], and social deprivation indices predict HF readmission independent of clinical severity [22]. The HEARTLAND Risk Score addresses this gap by incorporating distance to care and social support as supplementary variables alongside established clinical risk factors (Table 6). While the score requires formal validation - a planned next step - the rationale for including these variables is supported by robust observational evidence.

Distinguishing Evidence Strength

A key feature of this protocol is explicit labeling of evidence strength, a practice grounded in the 2022 AHA/ACC/HFSA guideline's own classification system [2]. SGLT2 inhibitors for HFrEF carry a Class I recommendation; for HFpEF, the recommendation is Class IIa per the 2022 AHA/ACC/HFSA guideline [2], with subsequent evidence from the EMPEROR-Preserved [6] and DELIVER [7] trials and expert consensus pathways supporting use across the LVEF spectrum. Finerenone for HFpEF represents emerging evidence from the FINEARTS-HF trial [8] that is promising but still being integrated into guidelines. The HEARTLAND Risk Score is a pragmatic heuristic designed for clinical decision support. This transparency is particularly important in rural settings where clinicians may have less access to continuing education and specialist consultation, making clear evidence labeling an essential safeguard against inappropriate extrapolation of emerging findings.

Low-Technology Validation

The Hozhó Trial's success with voice telephone calls is central to this protocol's design [13]. While conducted in a specific population - American Indians in the rural Navajo Nation - the principle that voice telephone-based optimization can achieve clinically meaningful improvements in GDMT prescribing has broad implications for rural communities facing similar constraints of limited broadband and digital literacy. Digital health solutions often assume connectivity and technological literacy that many rural patients lack, creating a paradox where the communities with the greatest need for innovative care delivery are least able to access technology-dependent solutions. By validating analog pathways as primary interventions rather than fallback options, the protocol ensures functionality across the digital divide. This approach aligns with the broader principle that healthcare innovations must be designed for their intended populations, not adapted after the fact from solutions designed for resource-rich environments.

Pharmacoeconomic Reality

Novel therapies, including sacubitril/valsartan, finerenone, and semaglutide, cost $400-1,000 or more monthly, which is prohibitive for many patients in the communities HEARTLAND targets. The "generic bridge" pathway ensures immediate treatment, while optimal agents are pursued through PAP and insurance navigation. This pragmatic approach - explicitly authorizing generic therapy as acceptable rather than inadequate - prevents therapeutic nihilism in cost-constrained settings. The pharmacoeconomic navigation framework recognizes that financial barriers to GDMT represent a systemic failure, not a patient failure, and provides structured tools for addressing cost as a modifiable barrier to optimal care.

Policy Alignment

The announcement of the CMS Ambulatory Specialty Model for 2027 [15] signals a federal commitment to value-based HF management that aligns with HEARTLAND's approach. The AHA's Presidential Advisory on Rural Health explicitly called for development of delivery models adapted to rural constraints [11], and the AHA Scientific Statement on Implementation Science for HF Equity identified the need for frameworks addressing geographic barriers [16]. HEARTLAND's tiered implementation structure, with its built-in quality metrics and outcome tracking, provides the infrastructure needed for value-based contracting while responding directly to these institutional calls to action.

Clinical Implications and Next Steps

For rural primary care providers, HEARTLAND offers an actionable pathway that can be initiated with existing resources. Although prospective validation is needed, the following steps represent an immediately actionable starting point grounded in established evidence. A PCP at a critical access hospital can implement Tier 1 with five foundational steps: calculate the HEARTLAND Risk Score at discharge, initiate at least two GDMT classes prioritizing SGLT2i and beta-blockers, establish a 48-72 hour post-discharge telephone call, provide condensed teach-back on three core domains, and enroll appropriate patients in the "generic bridge" pathway. These five steps require no specialist consultation, no digital infrastructure, and no additional staffing beyond the existing workforce with standardized scripts. As capacity builds, the facility can progressively adopt Tier 2 elements. The tiered structure means that perfect is not the enemy of good - incremental improvement from baseline constitutes success in settings where any systematic HF management represents an advance over current practice.

Limitations

Several limitations warrant acknowledgment. First, this is a targeted narrative review, not a systematic review with Preferred Reporting Items for Systematic Reviews and Meta-Analyses (PRISMA) methodology. We did not conduct a formal risk of bias assessment or a comprehensive database search. This approach is appropriate for an implementation framework but limits claims about comprehensiveness.

Second, the HEARTLAND Risk Score is a pragmatic heuristic without statistical validation. Weights were assigned based on relative effect sizes in the cited observational literature [3,4,21,22] rather than through formal derivation, and the score's discriminative ability and calibration have not been tested through derivation and validation cohorts. It should supplement validated instruments, not replace them.

Third, the integrated HEARTLAND bundle has not been prospectively tested. Effect sizes cited derive from component interventions tested individually, and the additive or synergistic effects of combining these interventions within a single protocol remain theoretical. Actual outcomes will depend on implementation fidelity, patient populations, and local context.

Fourth, while SGLT2i for HFpEF now carries a Class IIa recommendation supported by two landmark trials [6,7], other emerging evidence for HFpEF therapies - including finerenone and semaglutide - is still being incorporated into guidelines. Recommendations may evolve as additional data and guideline updates emerge, and clinicians should monitor for updated guidance from the AHA, ACC, and HFSA.

Fifth, the protocol was developed through literature review and evidence synthesis rather than direct participatory design with rural healthcare teams. While informed by publicly reported data from rural healthcare settings, prospective stakeholder engagement during pilot implementation will be essential to identify operational barriers not anticipated during protocol design.

Sixth, federal payment policy, specifically the CMS ASM anticipated for 2027, is subject to change [15]. Details presented reflect announced policy as of December 2025, and implementers should verify current policy status.

Future Directions

Several research priorities emerge from this work. First, pilot implementation studies in critical access hospitals and FQHCs are warranted to assess real-world feasibility and identify barriers not anticipated during protocol design. Second, formal validation of the HEARTLAND Risk Score through derivation and validation cohorts - potentially using GWTG-HF registry data linked with geographic and social determinant variables - would establish its discriminative ability and calibration characteristics, transforming it from a pragmatic heuristic into a validated instrument. Third, electronic health record (EHR)-integrated clinical decision support could automate risk stratification and titration reminders, reducing cognitive burden on clinicians in high-volume settings. Fourth, cost-effectiveness analysis across implementation tiers would inform resource allocation decisions and support value-based contracting applications. Fifth, the global burden of HF, affecting over 64 million people worldwide [27], underscores the potential international applicability of tiered implementation frameworks such as HEARTLAND, particularly in low- and middle-income countries facing similar challenges of specialist scarcity and resource variability.

## Conclusions

HEARTLAND provides a structured, tiered framework to extend evidence-based HF care to the millions of Americans living in communities without local cardiologist access. To our knowledge, it represents the first published implementation protocol specifically designed for primary care-led HF management in rural and resource-limited US settings, addressing a gap identified by professional societies and unmet by existing quality improvement programs and registries. The protocol's tiered design accommodates resource variability from critical access hospitals to regional referral centers, its dual-track system bridges connectivity gaps by validating both digital and analog monitoring pathways, and its "generic bridge" pathway ensures that cost barriers do not prevent treatment initiation.

Pharmacological recommendations are grounded in landmark trial evidence - including EMPEROR-Preserved and DELIVER trials for SGLT2i across the LVEF spectrum - with explicit differentiation between established, emerging, and pragmatic levels of evidence. The HEARTLAND Risk Score supplements established prognostic instruments by incorporating rural-specific variables - distance to care and social support - that current scores omit despite robust evidence of their prognostic relevance. Implementation science requires the balance of aspiration and realism. HEARTLAND is designed to function across resource levels - from settings with advanced technology and specialist support to those relying on telephone calls and paper diaries - while maintaining the scientific rigor and operational honesty essential for both academic credibility and real-world effectiveness.
